# The Role of Toll-like Receptors (TLRs) and Their Related Signaling Pathways in Viral Infection and Inflammation

**DOI:** 10.3390/ijms24076701

**Published:** 2023-04-04

**Authors:** Ralf Kircheis, Oliver Planz

**Affiliations:** 1 Syntacoll GmbH, 93342 Saal an der Donau, Germany; 2 Institute of Cell Biology and Immunology, Eberhard Karls University Tuebingen, 72076 Tuebingen, Germany; oliver.planz@uni-tuebingen.de

## 1. Introduction

Toll-like receptors (TLRs) belong to a powerful system for the recognition and elimination of pathogen-associated molecular patterns (PAMPs) from bacteria, viruses, and other pathogens. They are also involved in recognizing and eliminating self-derived, damage-associated molecular patterns (DAMPs) released from dying or lytic cells. Typical PAMPs are nucleic acids, including viral RNA and DNA, but they also include surface-exposed glycoproteins, lipoproteins, and various membrane components.

TLRs are highly expressed in immune cells, such as dendritic cells (DCs) and macrophages, as well as in non-immune cells, such as fibroblasts and endothelial cells. Activation of TLRs leads to the production of proinflammatory cytokines and type I interferons, which are important for induction of the host immune response against bacterial, fungal, and viral infections and malaria. However, dysregulation and overstimulation can be detrimental, leading to hyperinflammation, sepsis, and loss of tissue integrity. TLRs are involved in the pathogenesis of acute viral infections.

The common theme of this Special Issue is the role of toll-like receptors (TLRs) and their related signaling pathways in viral infection and inflammation, including sterile inflammation and inflammation in response to mechanical stress, tissue remodeling, and cancer. Four reviews and six experimental articles are published in this Special Issue. The reviews focus on the topics of viral infection-induced inflammation, such as SARS-CoV-2 [1,2] and hepatitis C virus (HCV) infection [3], related to TLR-induced pathological processes; they also discuss the protective role of TLRs against viral infection and the strategies used by viruses to inhibit TLR activation. Furthermore, the role of immunoregulatory and antiviral oligonucleotides is addressed [4]. The six experimental research papers cover topics ranging from TLR2 and TLR4 polymorphisms and susceptibility to bacterial sepsis [5]; decrease in endotoxin-induced activation of TLR4 signaling by xanthohumol-rich hop extract [6], advanced glycosylation end-products and TLR- induced changes in aquaporin-3 expression in mouse keratinocytes [7]; TLR4 signaling in periodontal ligament cells in response to mechanical compression [8]; identification of optimal TLR8 ligand by altering the position of 2′-O-ribose methylation [9]; to a breast cancer vaccine containing a novel TLR7 agonist showing antitumor effects [10].

The two facets of TLR activation in viral infection are well illustrated by two review articles focusing on the detrimental effects of massive activation of TLR and the antiviral protective effects of TLR activation in the context of SARS-CoV-2 infection [1,2]. Patients in critical stage show acute respiratory distress syndrome (ARDS) of the lungs; coagulopathies; multi-organ dysfunction correlating with massive cytokine and chemokine release; and distortion of the complement and coagulation system, which is mostly triggered by hyperactivation of innate immune receptors and their related pathways, such as NF-κB, JAK/STAT, and MAPK [1,2].

**Figure 1:**
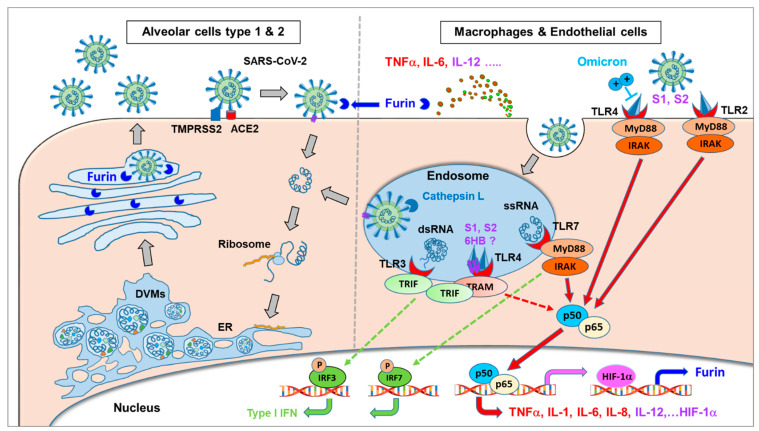
SARS-CoV-2 binds via the spike protein to ACE2, followed by fusion with the host cell membrane. Viral RNA transcription and translation of viral proteins occur in double-membrane vesicles (DMVs). The newly assembled virus particles leave the cells via the Golgi apparatus where the spike protein undergoes proteolytic cleavage by furin. This process occurs preferably in virus-producing cells with a high TMPRSS2 expression, such as alveolar cells (**left side**). Alternatively, the virus can be taken up into endosomes, predominantly in cathepsin L-rich cells, such as innate immune cells and endothelial cells (**right side**). Several components of the SARS-CoV-2 virus act as PAMPs by activating various TLRs, resulting in massive activation of the NF-kB (p50/p65) pathway. Different cells differ regarding their expression of TLRs, with high levels of TLR4 and TLR2 expressed by macrophages and endothelial cells, but low levels by alveolar lung cells. Adapted from [1].

The involvement of cell types that differ greatly in their TLR patterns and their pro-inflammatory potential will finally determine the level of pro-inflammatory activation. Whereas alveolar cells that highly express TMPRSS2 in the lungs are the main drivers of viral replication, the major pathophysiological effects are derived from over-activation of innate immune cells, such as macrophages and endothelial cells. In this context, the lower pathology (despite higher infectivity) of the omicron variant compared to previous VOCs has been hypothesized to correlate with a lower TLR- and NF-κB activation due to a changed charge distribution in the spike protein (Figure 1) [1].

**Figure 2:**
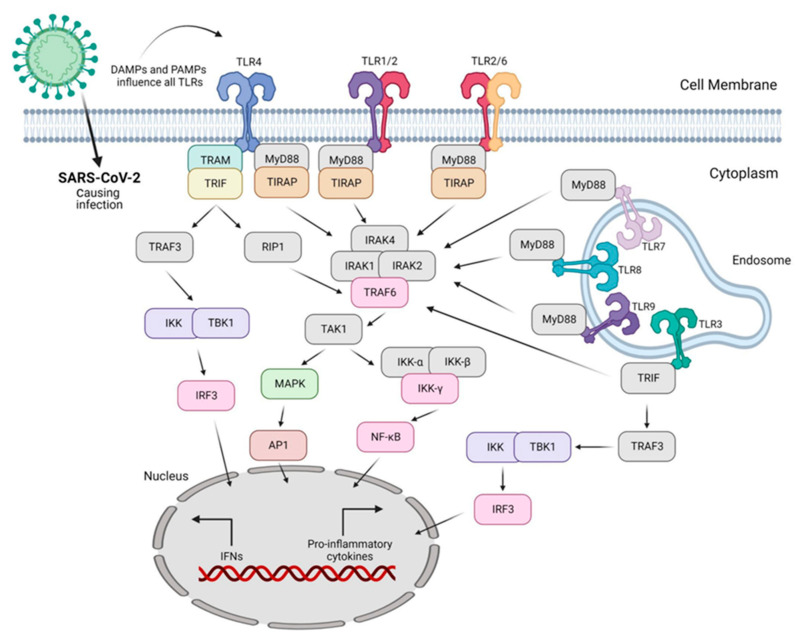
SARS-CoV-2 infection causes DAMPs and PAMPs that are recognized by a broad variety of toll-like receptors (TLRs), leading to the expression of pro-inflammatory cytokines and interferons. Reproduced from [2].

The second article discusses how the outcome of SARS-CoV-2 infection is dependent on the balance between induced antiviral immunity and tissue damage. Surface (TLR2 and 4) and intracellular (TRL3, 7/8, and 9) factors are involved in the recognition of SARS-CoV-2 infection by the immune system. Adaptors such as MyD88 and TRIF are recruited by TLRs to initiate the signaling pathways (Figure 2).

The nature of the ligand and downstream adaptor molecules direct the TLR signaling cascade into two distinct pathways, i.e., MyD88-dependent and -independent pathways. The MyD88-dependent pathway employed by all TLRs (except TLR3) triggers the activation of pro-inflammatory signal transduction pathways, such as NF-κB and mitogen-activated protein kinases (MAPKs), resulting in the release of inflammatory cytokines. In contrast, the TRIF-dependent pathway involved in TLR3 and TLR4 signaling leads to the stimulation of IRF3 expression of IFN-I, which primes the cells for antiviral activities. Expression of another important interferon regulatory factor, IRF7, is triggered by TLR7–9. The production of type I and type III IFNs by TLRs is a significant antiviral feature essential for systemic viral control and viral clearance [2].

**Figure 3:**
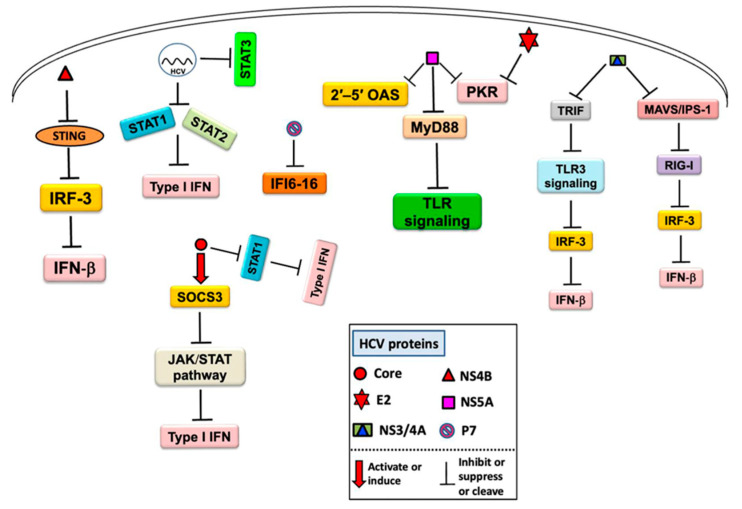
An overview of the mechanism of host innate immune response inhibition by HCV and its proteins. Reproduced from [5].

The dual role of TLR activation has also been shown for HCV infection. Protective effects have been shown for TLR3/4/7/8/9, whereas adverse effects have been demonstrated, e.g., for TLR4 and TLR8. The protective effects of TLR activation may potentially outbalance the adverse side effects in the case of chronic HCV infection. Accordingly, HCV has developed multiple strategies to inhibit the innate immune response and dispose the host toward chronic infection, largely via virus-derived TLR inhibitors (Figure 3).

A clear understanding of TLR interactions in HCV infection is critical for developing new therapeutic approaches to fight the disease, including TLR agonist-adjuvanted HCV vaccines [3].

**Figure 4:**
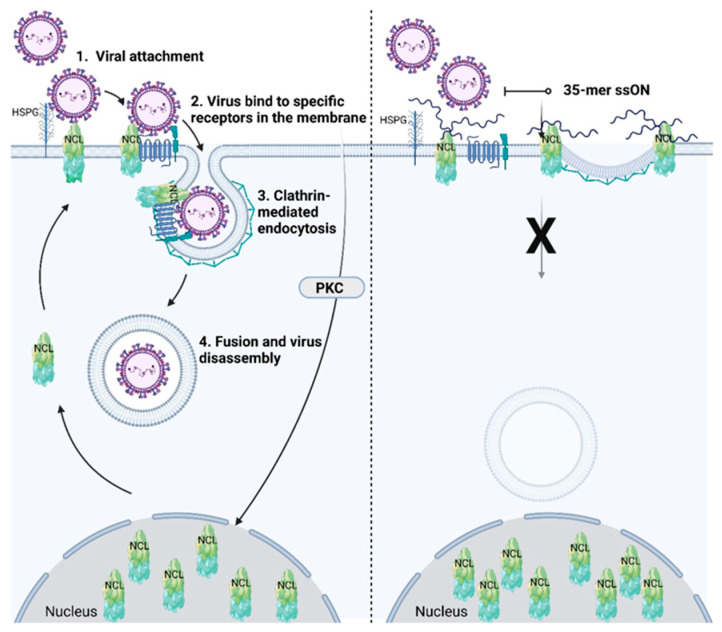
Schematic illustration of nucleolin (NCL) as a receptor for viruses and its participation in clathrin-mediated endocytosis (**left**), which can be inhibited by 35-mer ssON (**right**). Viruses attach to the cellular membrane using, e.g., heparan sulfate proteoglycan and/or NCL, followed by clathrin-mediated endocytosis. 35-mer ssON, but not 15-mer ssON, can inhibit viral attachment by shielding NCL. 35-mer ssON is hypothesized to confer steric hindrance for endocytosis. Reproduced from [4].

The fourth review discusses the role of immune regulatory and antiviral oligonucleotides regarding TLR-3, 7, 8, and 9, which are located within endosomes and sense oligonucleotides that are taken up into the endosomes. There seems to be an abundant pool of 30–40 oligonucleotides derived from different RNAs, such as tRNA, rRNA, and mRNA. This 30–40 oligonucleotide pool may confer a “buffering” system to regulate the uptake of endocytic cargo into cells. Some single-stranded oligonucleotides (ssONs) may have the capacity to inhibit endocytic pathways and have immunoregulatory functions. In addition, this pool of 30–40 oligonucleotide RNAs may also prevent the entry of certain viruses (Figure 4) [4].

Toll-like receptors (TLRs) play an eminent role in immune responses to bacterial pathogens, and are involved in the development of sepsis. TLR genetic variants might influence individual susceptibility to develop sepsis. In an experimental study, the association of genetic polymorphisms of TLR2 and TLR4 with the risk of developing sepsis was investigated based on single nucleotide polymorphisms (SNPs). The DNA samples of patients in intensive care unit were genotyped using RT-PCR technology. Significant associations between TLR2 Arg753Gln polymorphisms and sepsis and between TLR4 Asp299Gly polymorphisms and *Acinetobacter baumannii* infection were found. This study concludes that the TLR2 genotype may be a risk factor for sepsis in adult patients [5].

Infections with Gram-negative bacteria are among the leading causes of infection-related deaths. To find new therapeutic approaches to control bacterial infection-triggered sepsis, the effect of xanthohumol-rich hop extract on endotoxin-induced activation of TLR-4 signaling in human peripheral blood mononuclear cells was investigated. Previous studies had suggested that chalcone xanthohumol (XN) found in hop has anti-inflammatory effects. A placebo-controlled, randomized cross-over design study assessed if the oral intake of a single low dose of XN could affect lipopolysaccharide (LPS)-induced immune responses in peripheral blood mononuclear cells (PBMCs) ex vivo in normal-weight healthy women. LPS-dependent activation of hTLR4-transfected cells was significantly and dose-dependently suppressed by the XN-rich hop extract, which was attenuated when cells were co-challenged with sCD14. These results suggest that even low doses of XN consumed in a XN-rich hop extract can suppress LPS-dependent stimulation of PBMCs due to the interaction of the hop compound with the CD14/TLR4 signaling cascade [6].

The present Special Issue also covers the roles of TLRs in wound healing, after mechanical stress, and in cancer. Usually, inflammation is finally exchanged by processes restoring physiological homeostasis, such as fibrinolysis, wound healing, and re-epithelization. Prolonged inflammation and impaired re-epithelization are major contributing factors to chronic non-healing diabetic wounds. Advanced glycation end-products (AGEs) and the activation of toll-like receptors (TLRs) can trigger inflammatory responses. Aquaporin-3 (AQP3) plays an essential role in keratinocyte function and skin wound re-epithelialization, regeneration, and hydration. Suberanilohydroxamic acid (SAHA), a histone deacetylase inhibitor, mimics the increased acetylation observed in diabetes. The effects of TLR2/TLR4 activators and AGEs on keratinocyte AQP3 expression in the presence and absence of SAHA was studied in primary mouse keratinocytes. The results indicate that TLR2 activation and AGEs may be beneficial for wound healing and skin hydration under normal conditions via AQP3 upregulation, but these pathways are likely deleterious in diabetes [7].

The involvement of TLRs in sterile inflammation induced by mechanical stress on tissues was studied in another study. Mechanical compression by simulating orthodontic tooth movement in in vitro models induced pro-inflammatory cytokine expression in periodontal ligament (PDL) cells. Primary PDL cells were studied for cell signaling downstream of key molecules involved in the process of sterile inflammation via TLR4. The TLR4 monoclonal blocking antibody was found to reverse the upregulation of phospho-AKT caused by compressive force. Overall, this study provides evidence that TLR4 is involved in the modulation of sterile inflammation during mechanical stress, such as during orthodontic therapy and periodontal remodeling [8].

Recognition of RNA by TLRs is regulated by various posttranslational modifications. Different single 2′-O-ribose (2′-O-) methylations have been shown to convert TLR7/TLR8 ligands into specific TLR8 ligands. A study investigated whether the position of 2′-O-methylation is crucial for its function. An 18S rRNA-derived TLR7/8 ligand, RNA63, was found to be differentially digested as a result of 2′-O-methylation, leading to variations in TLR8 and TLR7 inhibition. The suitability of certain 2′-O-methylated RNA63 derivatives as TLR8 agonists was further supported by the fact that other RNA sequences were only weak TLR8 agonists. Specific 2′-O-methylated RNA derivatives were identified as optimal TLR8 ligands [9].

The last paper of this Special Issue addresses the use of TLR agonists as adjuvants for anti-cancer vaccination against mucin 1 (MUC1), a tumor-associated antigen that is highly expressed in breast cancer. The authors constructed a novel tumor vaccine (SZU251 + MUC1 + Al) containing MUC1 and two types of adjuvants, a TLR7 agonist (SZU251) and an aluminum adjuvant (Al). Immunostimulatory responses were first verified in vitro, showing that the vaccine promoted the release of cytokines and the expression of costimulatory molecules in mouse bone marrow dendritic cells and spleen lymphocytes. Importantly, SZU251 + MUC1 + Al was effective and safe against tumors expressing the MUC1 antigen in both prophylactic and therapeutic schedules in vivo. The immune responses in vivo were attributed to the increase in specific humoral and cellular immunity, including antibody titers, CD4^+^, CD8^+^, and activated CD8^+^ T cells. The results indicate that TLR agonists can be successfully used as new vaccine adjuvant candidates for the prevention and treatment of breast cancer [10].

## 2. Discussion

Overall, this Special Issue illustrates the two sides of TLRs as drivers of pathogenesis of acute bacterial and viral infections, including COVID-19, but also as potent players for antiviral, antibacterial, and anti-cancer immune activation. Dependent on the direction of the disbalance in TLR activation in different pathologies, TLR agonists [9,10] or antagonists [2,4,6] may be of interest. For stimulating innate immune responses against bacterial, viral, or parasitic invasions or for use as an adjuvant in anti-cancer vaccination, one single selected TLR agonist may be sufficient to trigger an effective innate immune response. In contrast, inhibiting the hyperactivation of the innate system can be much more complex due to the involvement of multiple and diverse TLRs in various pathological conditions [1,3]. Therefore, it will be difficult to choose one TLR antagonist to inhibit all TLRs that may be involved in the induced systemic hyperactivation. Moreover, secondarily induced cytokines, such as TNFα, IL-1, and IL-6, will activate additional receptors, amplifying the immune stimulatory signaling [1,11].

In order to inhibit the entire immune stimulatory signaling cascades induced, e.g., by life-threatening acute viral infections (such as Ebola, dengue fever, SARS-CoV-2, and RSV) [1,2,12] or by bacterial sepsis [5], inhibition of **(i)** specific downstream adaptor molecules or of **(ii)** central signaling pathways may be more specific to obtain the desired effect or to provide a more generalized modulation of signaling, respectively. Regarding downstream adaptor molecules, the MyD88-dependent pathway (involved in the signaling of all TLRs, except for TLR3) primarily triggers the activation of pro-inflammatory signal transduction pathways, such as NF-κB and mitogen-activated protein kinases (MAPKs) [1,2,3]. In contrast, the TRIF-dependent pathway involved in TLR3 and TLR4 signaling leads to the stimulation of IRF expression of IFN-I, which primes the cells for antiviral activities [2]. Specific inhibition of Myd88 signaling without affecting TRIF-mediated signaling may inhibit the expression of pro-inflammatory cytokines, with no (or only marginal) effect on IRF expression. Furthermore, secondary adaptor molecules, such as TRAF3 and TRAF6, may provide additional levels for modulating the ratio between the induction of IRF and pro-inflammatory signaling pathways after TLR activation [13].

To attenuate TLR signaling, the inhibition of whole signal transduction pathways, such as NF-κB [1] or JAK/STAT [11], may provide a powerful modality to control the multiple additive, synergistic, triggering, and amplifying signaling cascades that have been induced during life-threatening acute viral infections [1,11].

## 3. Conclusions

TLRs are the drivers of the pathogenesis of acute bacterial and viral infections, but they are also essential for antiviral, antibacterial, and anti-cancer immune activation. Accordingly, TLRs, including their molecular adaptors and related signaling transduction pathways, are promising targets for pharmacological intervention and treatment.
